# Kinetics of tau aggregation reveals patient-specific tau characteristics among Alzheimer’s cases

**DOI:** 10.1093/braincomms/fcab096

**Published:** 2021-05-04

**Authors:** Tarun V Kamath, Naomi Klickstein, Caitlin Commins, Analiese R Fernandes, Derek H Oakley, Matthew P Frosch, Bradley T Hyman, Simon Dujardin

**Affiliations:** 1 Alzheimer Research Unit, Department of Neurology, Massachusetts General Hospital, Charlestown, MA 02129, USA; 2 Harvard Medical School, Boston, MA 02115, USA; 3 C.S. Kubik Laboratory for Neuropathology, Massachusetts General Hospital, Boston, MA 02129, USA

**Keywords:** tau, seeding, kinetics, propagation, heterogeneity

## Abstract

The accumulation of tau aggregates throughout the human brain is the hallmark of a number of neurodegenerative conditions classified as tauopathies. Increasing evidence shows that tau aggregation occurs in a ‘prion-like’ manner, in which a small amount of misfolded tau protein can induce other, naïve tau proteins to aggregate. Tau aggregates have been found to differ structurally among different tauopathies. Recently, however, we have suggested that tau oligomeric species may differ biochemically among individual patients with sporadic Alzheimer disease, and have also showed that the bioactivity of the tau species, measured using a cell-based bioassay, also varied among individuals. Here, we adopted a live-cell imaging approach to the standard cell-based bioassay to explore further whether the kinetics of aggregation also differentiated these patients. We found that aggregation can be observed to follow a consistent pattern in all cases, with a lag phase, a growth phase and a plateau phase, which each provide quantitative parameters by which we characterize the aggregation kinetics. The length of the lag phase and magnitude of the plateau phase are both dependent upon the concentration of seeding-competent tau, the relative enrichment of which differs among patients. The slope of the growth phase correlates with morphological differences in the tau aggregates, which may be reflective of underlying structural differences. This kinetic assay confirms and refines the concept of heterogeneity in the characteristics of tau proteopathic seeds among individuals with Alzheimer’s disease and is a method by which future studies may characterize longitudinal changes in tau aggregation and the cellular processes which may influence these changes.

## Teaser

In a reporter assay, Kamath et al. report differences in the kinetics of tau aggregation accross individuals in brain samples from sporadic Alzheimer patients reflecting the properties of the initial seeds and suggesting person-specific differences in tau pathobiology even among individuals with sporadic Alzheimer disease.

## Graphical Abstract

**Graphical Abstract:**
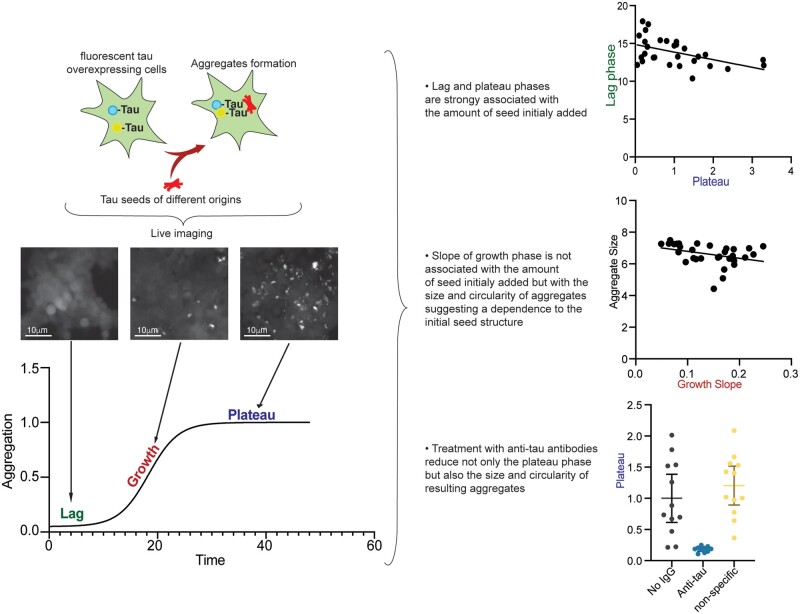

## Introduction

The aggregation of tau protein is a common histopathological marker shared by a number of neurodegenerative conditions called tauopathies – these include Alzheimer’s disease, Pick’s disease and progressive supranuclear palsy.^1^ Despite the prevalence of these tauopathies, the mechanisms of tau aggregation are still poorly understood. Many tauopathies show a characteristic, stereotyped spatial distribution of tau aggregates, one which is unique to the disease but is shared across those with the same condition.^2–6^ In addition, inoculation of exogenous tau fibrils into *in vivo* and *in vitro* models of tauopathy leads to aggregation phenotypes reflective of properties of the initial tau aggregates.^7–11^ Aggregates themselves vary significantly both structurally and biochemically across tauopathies, indicating the potential presence of tau ‘strains’ that may mediate the diversity of clinical symptoms associated with these disorders.^11–16^

We recently suggested that within a population with sporadic Alzheimer’s disease, individuals appear to have tau that can be heterogeneous in biochemical properties and in its ability to initiate aggregation.^17^ We reasoned that this process can be considered to be a product of a set of discrete steps: (i) uptake of tau seeds; (ii) delivery of the seeds to the cytoplasm; (iii) interaction of the seeds with the endogenous tau protein (or peptide produced by the reporter cell line); and (iv) formation of an aggregate. Each of these steps are more or less characterized in the literature^18^ and are likely to have separate kinetics. To study these steps and investigate the kinetics of the tau aggregation and propagation process, we have adapted a well-established tau aggregation biosensor assay^8^ to continuously monitor the cells using an *in vitro* imaging chamber to allow direct observation of the kinetics of tau aggregation. These cells are advantageous in that, unlike neurons, they do not take up tau unless presented with a protein transduction reagent such as lipofectamine, thereby simplifying the problem of analysing the kinetics of each step by eliminating steps 1 and 2. Moreover, this stable cell line contains a discrete amount of bioreporter CFP/YFP pairs in each cell, thus eliminating step 3 as a source of variability across cases. Finally, tau strains can be reproducibly identified using the morphology of formed inclusions.^10^^,^^11^ Thus, we were able to monitor the kinetics of tau aggregation with a focus on the rate and extent to which different tau seeds generate aggregates in a cellular context.

We find that aggregation kinetics follow an expected logistic pattern of growth in number of aggregates with phases related to both the amount of seed-competent tau species and the structural properties of the initial seeds. The current data confirm that these parameters differ across a panel of individual patients with Alzheimer’s disease, who are differentiated from one another also by the relative aggressiveness of their rates of clinical progression. Indeed, more aggressive seeding is correlated with a more aggressive clinical course,^17^ in accordance with the idea that the rate of propagation across neural systems, which may rely in part on seeding properties, correlates with the rate of progression.^19^ In all, our experiments dissect some of the intracellular molecular processes that give rise to a characteristic kinetic seeding shape as well as inform on what influences tau aggregation.

## Materials and methods

### Human tissue collection and homogenization

Alzheimer’s disease subjects post-mortem brains were selected from the Massachusetts Alzheimer’s Disease Research Center Longitudinal Cohort study based on several criteria: (i) Clinically diagnosed with dementia due to probable Alzheimer’s disease; (ii) Cognitive status assessed at least 3 times by an MGH neurologist and scored using the Clinical Dementia Rating—Sum Of Boxes scale; (iii) Diagnosis of Alzheimer’s disease confirmed postmortem by an MGH neuropathologist; (iv) Braak Neurofibrillary tangles stage V or VI as determined by total tau immunostaining and silver staining as previously described^20^; and (v) the least possible concurrent neuropathologies. Information regarding the subjects whose brains were used, including the sex, age of onset, age at death and Apolipoprotein E genotype were also collected and are indicated in Table 1.

**Table 1:** Demographic characteristics for patient cases of sporadic Alzheimer’s disease. Included below are the following characteristcs: the age of the subject at the onset of sporadic Alzheimer’s disease, the age of the subject at death and the maximum Clinical Dementia Rating-Sum of Boxes score of the subject. Subjects with possibly alternative neuropathologies and interfering factors were excluded from the student, and all patients showed the highest level of tau propagation at death (i.e. Braak tau neurofibrillary tangle stage V/VI).

Alzheimer’s disease Case	Sex	Age of onset	Age at death	Apolipoprotein E genotype
Positive control	M	Not reported	91	e3/e4
1	M	80	99	e3/e3
2	M	66	73	e3/e4
3	F	86	95	e3/e4
4	F	78	95	e3/e4
5	M	75	92	e3/e4
6	M	65	76	e3/e4
7	F	77	85	e3/e4
8	F	82	91	e3/e4
9	M	68	86	e3/e3
10	M	72	85	e3/e4
11	M	80	89	e3/e3
12	M	74	84	e3/e4
13	F	59	64	e3/e4
14	M	58	67	e3/e2
15	F	75	87	e3/e4
16	M	74	86	e3/e4
17	M	64	82	e3/e4
18	M	80	89	e3/e3
19	M	66	77	e4/e4
20	M	81	90	e3/e3
21	F	65	75	e4/e4
22	M	57	67	e3/e4
23	F	49	60	e3/e3
24	M	64	81	e3/e4
25	M	68	84	e3/e4
26	M	66	72	e3/e3
27	F	50	58	e3/e4
28	M	68	76	e3/e4
29	M	68	84	e4/e4
30	F	70	83	e4/e4
31	M	70	84	e4/e4

Sampling of human tissue was previously described,^21^ but in short, brains were separated into two hemispheres. One of the hemispheres was sliced coronally at the autopsy and 1 cm-thick slabs were flash frozen and stored at −80°C. At a later time, approximately 1 g of frontal cortex was dissected out of the frozen brain section corresponding to Brodmann’s Area 8/9 and stored at −80°C until homogenization.

Frozen human tissue was thawed on wet ice until and then homogenized by hand dounce homogenization in 500 μL of PBS with 1× protease inhibitor (Roche) in a 2 mL glass dounce homogenizer with 30 up/down strokes. Homogenates were centrifuged at 10 000 × *g* for 10 min at 4°C and the supernatant was collected and aliquoted. To measure total protein concentration and total tau concentration, a bicinchoninic acid assay (Thermo Scientific Pierce) and an Enzyme-Linked Immunosorbent Assay (ELISA, Meso Scale Diagnostics) were performed, respectively, following the manufacturer’s protocol. For mouse brain tissue, homogenates were centrifuged at 3000 × *g* for 10 min at 4°C and the supernatant was collected and aliquoted.

### Seeding assay—*in vitro* Förster resonance energy transfer based assays

The *in vitro* Förster resonance energy transfer (FRET) based seeding assay has previously been described in detail^8^^,^^22^^,^^23^ and thus we will abbreviate the description herein. HEK293 cells were previously engineered to stably express the tau repeat domain region with the P301S mutation linked to familial frontotemporal dementia^24^^,^^25^ and tagged with either cyan fluorescent protein (CFP) or yellow fluorescent protein (YFP). Cells were cultured to around 70% confluence at 37°C, 5% CO_2_, in Dulbecco’s Modified Eagle Medium (ThermoFisher Scientific) supplemented with 10% fetal bovine serum and 1% penicillin and streptomycin. For subsequent use in the seeding assay, cells were plated at 40,000 cells per well in a black 96-well plate (Costar) coated with poly-d-lysine for adherence. Brain extract was then added 24 h later at a concentration of 8 ng of total tau per well. Extract was incubated in a 1% (v/v) Lipofectamine 2000 solution in OptiMem™ at room temperature for 10 min before being added to the wells at a total of 50 µL per well. For testing antibody reduction, 1 µg of antibody was incubated with the tau seeds prior to transfection for 15 min at 37°C. Tau aggregation was then quantitatively analysed either using flow cytometry using gating protocols as described previously^22^ or cytation live-cell imaging and subsequent image analysis.

#### Flow cytometry analysis of tau seeding

After incubating cells for 24 h with tau seeds, OptiMem™ was aspirated off of the cells and the cells were incubated with 50 µL of 1× trypsin-ethylenediaminetetraacetic acid (ThermoFisher Scientic) at 37°C for 7 min, and tapped periodically throughout. After trypsinization, 150 µL of cold Dulbecco’s Modified Eagle Medium supplemented with 10% fetal bovine serum and 1% penicillin and streptomycin was added to each well and the contents of each well was transferred to clear 96-well round-bottom plates (Corning). Cells were pelleted at 1500 × *g* for 10 min and media was removed. Cells were then fixed in a 2% (v/v) paraformaldehyde (Fisher Scientific) in phosphate buffered saline (PBS) for 10 min at room temperature in the dark. Cells were then pelleted once again at 1500 × *g* for 10 min and resuspended in PBS. Cells were run on a MacsQuant VYB flow cytometer (Miltenyi Biotec). Cells were initially gated on forward and side scatter, then were excited with a 405 nm laser. FRET and CFP fluorescence were read using 405/50 and 525/50 nm filters, respectively, and gated on being both YFP and CFP positive. Quantification of FRET signal was generated by creating a plot of CFP donor signal versus FRET signal, and cells which received the negative control brain extract were used to set boundaries for the FRET-negative population. The aggregation value was then reported as the integrated FRET density, defined as the percentage of FRET-positive cells multiplied by the median CFP fluorescence intensity.

#### Live-cell imaging analysis of tau seeding

After adding brain extract to the cells, the plate of cells was transferred to a Cytation 5 Multi-Mode Reader (BioTek), kept at 5% CO_2_ and 37°C, and imaged using the Gen5 Image Prime 3.03 Software. Four Green Fluorescent Protein images were taken of the cells in each well every half-hour for 72 h using a 20× objective lens. Images were exported from the Cytation 5 Imager and analysed using Fiji/ImageJ, in the Weka Segmentation package. Before full analysis, seven representative images were manually classified in a blind-manner to sort the features of each image into either cells, aggregates, or background. These seven images were then used to define an image segmentation model using the FastRandomForest classifier (a multi-threaded version of the Random Forest classifier) along with the following smoothing and edge-sharpening algorithms for pre-processing (as defined in the Weka Segmentation package): Gaussian blur, Hessian, Mean, Maximum, Variance, Minimum, Median, Laplacian and Entropy. The segmentation jobs were then exported to the Harvard Medical School Research Computing Cluster, O2. After segmentation, images were converted into 8-bit greyscale images with different pixel values reflecting one of the three features into which the image was classified (cells, aggregates or background). Aggregate number and size were then quantified using the Fiji/ImageJ Analyze Particles method and exported to Microsoft Excel. The aggregate count was fit to a logistic curve using the 4PL, X is log(concentration) function in GraphPad Prism, and the magnitude of the plateau phase of this curve was approximated as the average of the value of the logistic curve in the last 12 h of the experiment (the average number of aggregates present in the last 12 h), and used to normalize all seeding curves shown. We defined the slope of the growth phase (the steepness of the increase in the aggregate count) as the value of the Hill slope of the logistic curve fit to the data. Case #6 was excluded from the slope analysis as we were unable to fit a logistic curve.

### Statistical analysis

Basic statistics and plots have been generated using GraphPad Prism 7 (GraphPad). Unless otherwise stated, two-tailed Spearman *r* non-parametric correlations were used to correlate different variables obtained from single individuals. The Spearman correlation coefficient *r* and the *P*-value are indicated in the text and/or figure legends. For group comparisons, Unparied *t*-tests were used. *P*-values are indicated in the text and/or figure legends. To rule out potential effect of outliers on the overall results, we performed a sensitivity analysis by removing potential outliers and reapplying the Spearman *r* non-parametric correlations and saw no change. We therefore did not exclude any samples.

### Data availability

All data reported in this manuscript, are stored at the Massachusetts General Hospital, and are available from the corresponding authors upon reasonable request and with respect to the human research Institutional Review Board protocols.

## Results

**Figure 1:**
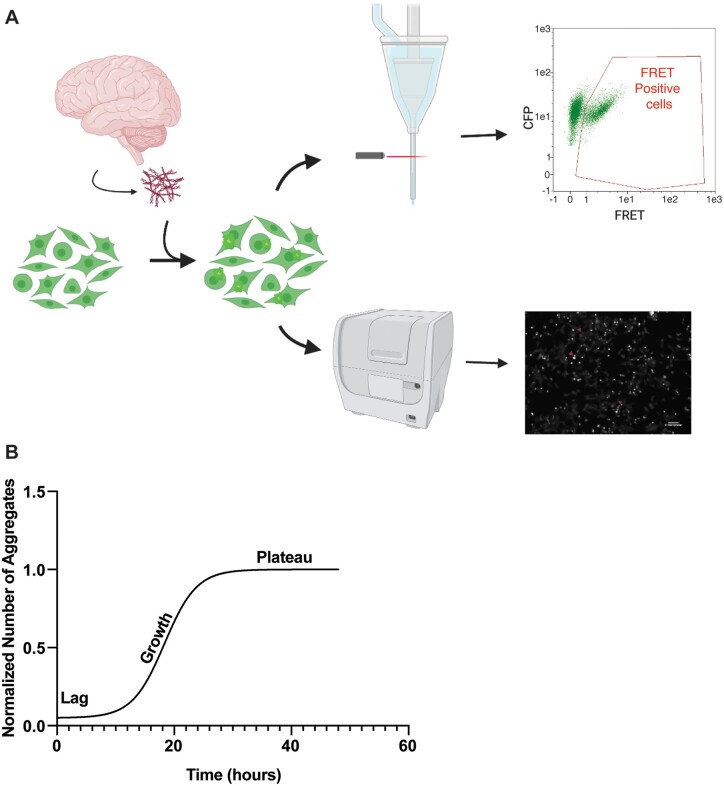
**Typical seeding assay diagram and kinetic results.** (**A**) Diagram representation of the tau biosensory seeding assay. HEK293 cells stably express the tau repeat domain either fused to cyan fluorescent protein (CFP) or yellow fluorescent protein (YFP). When cells are confluent, brain extract with tau seeds are transfected into the cells, forming bright aggregate punctae which are FRET-positive with a CFP donor and YFP acceptor fluorophore. This FRET signal is then detectable either by flow cytometry, leading to a shift in YFP signal or via live-cell imaging as punctae under a Green Fluorescent Protein filter which can be analysed. Diagram was created with Biorender.com. (**B**) Typical kinetic seeding curve for tau aggregation in the tau biosensor assay using tau seeds from a human case of sporadic Alzheimer’s disease. Tau aggregation follows a logistic growth, with a lag phase lasting 0–10 h, an exponential rise in the number of aggregates or growth phase lasting from 10 to 18 h, and a plateau phase during which the number of aggregates stays roughly constant from 18 h until the end of the kinetic imaging experiment. Images were taken once every half hour for 48 h and then analysed.

The tau biosensor assay is a useful technique to measure the tau seeding activity of a given biological sample, presumably driven solely by the properties of the tau aggregates in the sample.^8^ In brief, HEK293 cells stably overexpressing the microtubule binding region of tau with a P301S mutation, tagged with either CFP or YFP are cultured and incubated with brain extracts containing tau seeds from post-mortem Alzheimer’s disease patients as previously described.^8^^,^^21^^,^^26^ The formation of intracellular aggregates is detected and quantified by flow cytometry taking advantage of the energy transfer between the CFP and YFP, typically 24 h after inoculation with seeds. The use of flow cytometry provides an absolute quantification of all the aggregates in a well and provides accurate fluorescence intensity data in a high throughput manner.^8^^,^^21^ To resolve the time-course and kinetics of tau reporter aggregation in the biosensor assay, we instead used a Cytation 5 *in vitro* imaging platform, imaged the cells for 48 h, and developed an image analysis pipeline to analyse the images to count aggregates and assess their morphology (Fig. 1A).

**Figure 2:**
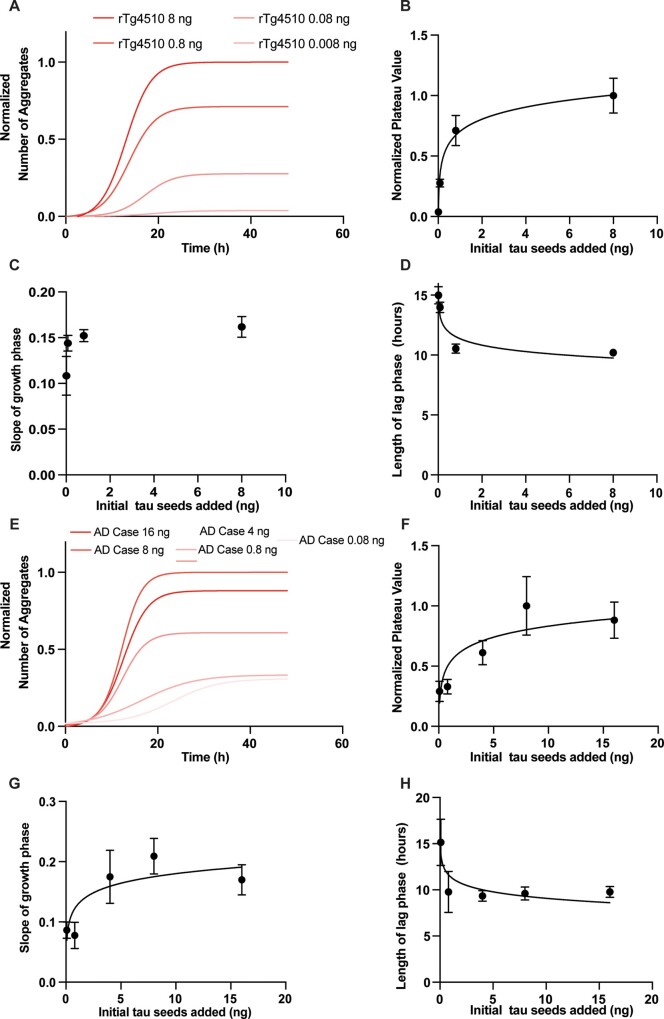
**Characteristics of tau seeding using increasing concentrations of tau seeds.** (**A**) The initial concentrations of tau seeds from the brain homogenate of an rTg4510 mouse was increased in a dose-dependent fashion, leading to a parallel increase in the number of aggregates which have formed by the plateau phase of the kinetic seeding curve, while maintaining the consistent logistic progression. (**B**) The plateau values of the non-linear regression of the seeding curves for seeding induced by different doses of inoculating rTg4510 tau seeds was plot against the concentration of the initial total protein in brain homogenate added. The plateau increases steeply at first and then stays relatively constant, resembling a logarithmic regression (*R*^2^ = 0.8102). (**C**) The slope of the growth phase of the non-linear regression of the seeding curves for seeding induced by different doses of inoculating rTg4510 tau seeds was plot against the concentration of the initial total protein in brain homogenate added. The slope values as a function of inoculating seed concentration resembled a logarithmic curve to a lesser degree than the plateau phase magnitudes (*R*^2^ = 0.3601) (regression not shown on the plot itself). (**D**) The lag phase length of the non-linear regression of the seeding curves for seeding induced by different doses of inoculating rTg4510 tau seeds was plot against the concentration of the initial total protein in brain homogenate added. The lag phase decreases at first then stays relatively constant, resembling a logarithmic regression (*R*^2^ = 0.7743). (**E**) The initial concentrations of tau seeds from the brain homogenate of a previously investigated human case of sporadic Alzheimer’s disease^31^ was increased in a dose-dependent fashion, leading to a parallel increase in the number of aggregates which have formed by the plateau phase of the kinetic seeding curve, while maintaining the consistent logistic progression. (**F**) The plateau values of the non-linear regression of the seeding curves for seeding induced by different doses of inoculating sporadic Alzheimer’s disease tau seeds were plot against the concentration of the initial total protein in brain homogenate added. The plateau increases steeply at first and then stays relatively constant, resembling a logarithmic regression (*R*^2^ = 0.5195). (**G**) The slope of the growth phase of the non-linear regression of the seeding curves for seeding induced by different doses of inoculating sporadic Alzheimer’s disease tau seeds was plot against the concentration of the initial total protein in brain homogenate added. The slope values seem to somewhat resemble a logarithmic regression (*R*^2^ = 0.4191) in which the slope values increase at first but then stay constant for all the other conditions (regression not shown on the plot itself). (**H**) The lag phase length of the non-linear regression of the seeding curves for seeding induced by different doses of inoculating sporadic Alzheimer’s disease tau seeds was plot against the concentration of the initial total protein in brain homogenate added. The lag phase decreases at first then stays relatively constant, resembling a logarithmic regression (*R*^2^ = 0.4976). All the data in this figure represent the average of four independent experimental replicates. Error bars represent s.d. of the mean.

The aggregates formed in the biosensor assay are readily detected and segmented in this assay format (Video 1). We first measured the time-course of aggregation and aggregation kinetics in this biosensor assay using brain homogenate taken from the frontal cortex of the rTg4510 mouse model,^27^^,^^28^ which overexpresses tau with the P301L mutation, a very similar mutation to that which already exists in the tau construct expressed by the biosensor cell line (P301S). We found that the tau aggregate number followed a logistic growth process characterized by an initial ‘lag’ phase in which there are very few aggregates, an exponential rise in the number and size of aggregates or ‘growth’ phase, after which the number of aggregates stays constant in a ‘plateau’ phase (Fig. 2A), a similar shape to what has been recorded in previous studies using alternative methods of measuring the time course of the protein aggregation.^8^^,^^29^^,^^30^

We then asked whether each of these phases (lag phase, growth phase and plateau phase) are concentration-dependent or -independent processes. We varied the concentration of total tau from the rTg4510 brain homogenate added to the biosensor cell line and found that the same logistic kinetic seeding curve was maintained across all conditions (Fig. 2A). Simple inspection reveals that the increase in initial aggregate concentration led to a parallel increase in the plateau phase to a ceiling, resembling a logarithmic function (Fig. 2A and B, *R*^2^ = 0.8102), suggesting that the observed plateau in aggregate count is not a simple depletion of the reporter tau repeat domain construct produced by the biosensor cells, but rather (at a low inoculating tau seed concentration) a function of the initial concentration of tau seeds added to the assay and of the ‘bioactivity’ of total tau protein added to initiate aggregation. It is worth noting that at very high concentrations the tau biosensor system *can* saturate, with little change in the plateau phase at higher inoculating tau seed concentrations, though it is unclear if this is due to the exhaustion of the reporter itself or the saturation of other cellular mechanisms.

In addition to the overt increase in the magnitude of the plateau phase, we investigated the effect of increasing tau seed concentration on the slope of the growth phase and length of the lag phase. We defined the slope of the growth phase (the steepness of the increase in the aggregate count) as the value of the Hill slope of the logistic curve fit to the data. We found only small differences in the slope of the growth phase with different concentrations of initial seeds, and that little of the variance in the slope of the growth phase was explained by differences in the tau seed concentration (Fig. 2C, *R*^2^ = 0.3601). We then approximated the length of the lag phase by calculating the time from *t* = 0 until the aggregate number reached 25% of the end average aggregate count. With increasing tau seed concentration, there was a parallel decrease in the length of the lag phase, to a floor, also resembling an inverse logarithmic curve (Fig. 2D, *R*^2^ = 0.7743). Overall, this suggests that, to a first approximation, the growth phase is a zero-order step to the seeding process, which is independent of the concentration of tau seeds, while the plateau phase magnitude and lag phase length are both concentration dependent processes.

We then tested a brain homogenate containing tau seed from a human autopsy sample of a patient with sporadic Alzheimer’s disease.^31^ Similar to the results with the rTg4510 tau seeds, when we varied the dose of inoculating tau seeds the magnitude of the plateau phase changed accordingly, until a ceiling, resembling a logarithmic curve (Fig. 2E and F). Although the slope of the growth phase increased slightly with an increase in the inoculating tau seed concentration, seed concentration did not strongly correlate with the slope of the curve (Fig. 2G). Finally, we found that, as with the rTg4510 tau seeds, the length of the lag phase was inversely correlated in a logarithmic manner with the dose of inoculating tau seeds (Fig. 2H).

**Figure 3:**
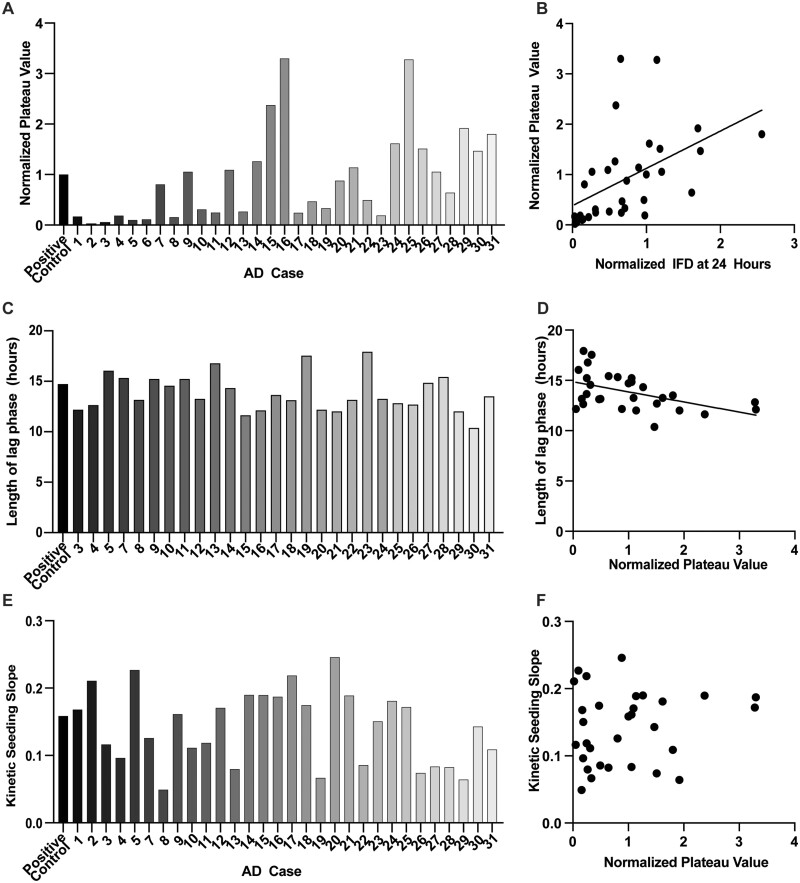
**Seeding is heterogeneous in kinetic characteristics across cases of sporadic Alzheimer’s disease.** (**A**) Tau seeding and aggregation were quantified via live-cell imaging and subsequent image analysis in all 32 Alzheimer’s disease cases. Samples were normalized to total tau levels, measured via MSD ELISA, before being added to the biosensor assay. After initial seeding, images were taken every half hour for 48 h. The number of aggregates was normalized to the positive control (kinetic seeding curves not pictured here, see Dujardin et al.^17^) Plateau phase magnitudes were normalized to the plateau phase magnitude for the positive control case, where the positive control was a previously well characterized Alzheimer’s disease Braak VI stage brain.^31^ (**B**) The average normalized integrated FRET density was plotted against the average number of aggregates counted in the last 12 h of kinetic imaging and a significant correlation was found between the two metrics for aggregation (*r* = 0.495, *P* = 0.0039). (**C**) The length of the lag phase of the kinetic seeding curve varied between patient cases. (**D**) The length of the lag phase of the kinetic seeding curve was plot against the plateau phase magnitude. There was a significant negative correlation between the plateau phase magnitude and the length of the lag phase of the kinetic seeding curve, indicating that higher seeders begin seeding at an earlier time point (*r* = 0.487, *P* = 0.0074). (**E**) The slope of the kinetic seeding curve or the rate of seeding, measured as the Hill Slope in the non-linear regression which was fit to the data, varied between patient cases. (**F**) The slope of the kinetic seeding curve was plot against the plateau phase magnitude and there was no significant correlation between the magnitude of the plateau phase (the metric for seeding activity) and the slope of the growth phase (the metric for rate of seeding) (*r* = 0.15, *P* = 0.4201).

We next tested how these findings could be expanded to explore the properties of tau aggregates across different human patients. We previously studied the clinical and pathological characteristics of a cohort of patients with Alzheimer’s disease and found a marked heterogeneity of tau seeding in this same biosensor line which was associated both with specific molecular characteristics of tau oligomers as well as differences in clinical disease progression.^17^ Therefore, we studied the kinetic of seeding in the ‘typical’ end-stage clinical and histopathological Alzheimer’s disease patients from this study (Table 1, *n* = 31). Importantly, in this experiment, the amount of total tau protein (8 ng of total tau as measured by ELISA) used to inoculate the tau biosensor cells was equivalent in each of the patient cases of sporadic Alzheimer’s disease. We observed that the profile of aggregation induced by seeds from all of these subjects followed the same general kinetic aggregation profile of logistic growth. However, the number of aggregates in the plateau phase varied from case-to-case (Fig. 3A) and correlated strongly with data obtained at 24 h on the flow cytometer (*P* = 0.0039, Fig. 3B), confirming the validity of the plateau phase measurement and longitudinal approach.

Notably, we found a significant negative correlation between the length of the lag phase of each sporadic Alzheimer’s disease kinetic seeding curve and the magnitude of the plateau phase of that case, with a significant non-zero slope (*P* = 0.0074, Fig. 3C/D). This analysis excluded those few cases for which the lag phase analysis could not be conducted since the value of the aggregate count cutoff used approached the noise of the assay itself (*n* = 3). This clearly suggests that those cases that had a higher seeding activity were also the ones that began seeding earlier in the assay, potentially due to a parsimonious cause, such as a higher concentration of oligomeric, seeding-competent tau per total tau protein in the ‘higher-seeding’ cases.^17^

In addition to the magnitude of the plateau phase, the slope of the growth phase also varied among subjects. In order to understand whether this observation is a simple function of the magnitude of the plateau phase or driven by differences in the ‘bioactivity’ or molecular characteristics of the tau seeds, we normalized the aggregation profiles of each case of sporadic Alzheimer’s disease to the average number of aggregates in the plateau phase. We found that there was variation in the slope of the exponential increase among cases (Fig. 3E) and, importantly, that there was no significant correlation between the slope of the normalized seeding curve and the magnitude of the plateau phase of a seeding curve for a given patient case (i.e. the seeding activity) (*P* = 0.4201, Fig. 3F), indicating that speed of aggregation *across patient cases* is a distinct parameter from the number of seeds, and that this differs across patients.

**Figure 4:**
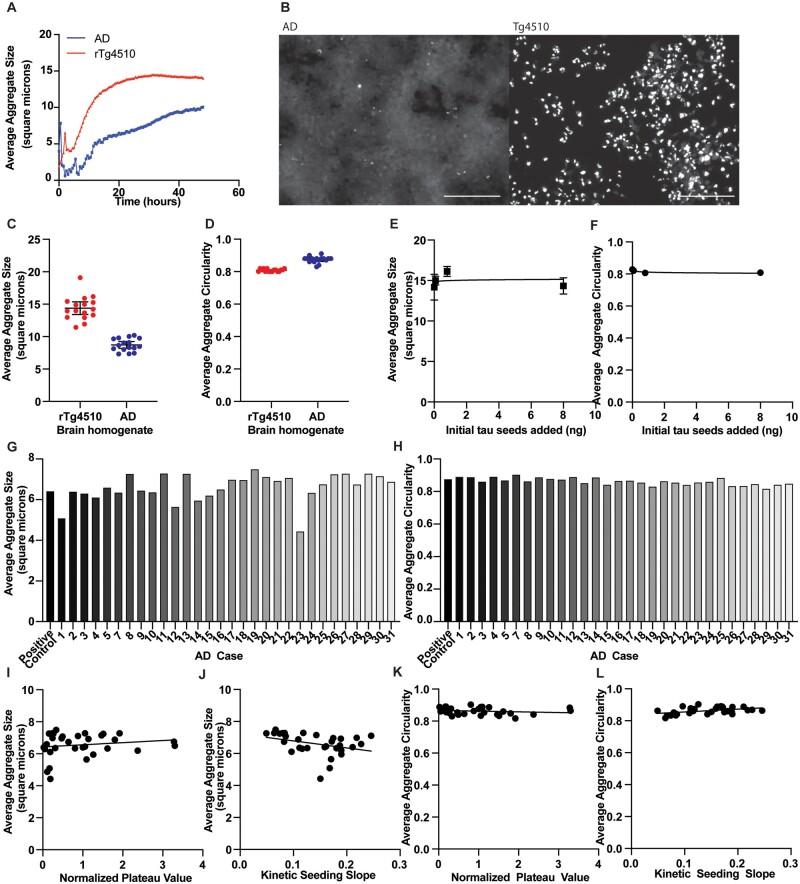
**Tau seeding morphology analysis.** (**A**) In both human and mouse brain extract seeding assays, the average aggregate size increases during the first part of the seeding assay, through the lag phase and growth phases, and then is more constant during the plateau phase, particularly in the last 12 h. *n* = 1 human subject and *n* = 1 rTg4510 mouse brain. This experiment was independently replicated three times with reproducible results. (**B**) Aggregate morphology is overtly different when aggregation is initiated using protein from the brains of those with sporadic Alzheimer’s disease (left) versus protein from rTg4510 mouse brains (right). Scale bar = 200 µm. (**C**) Aggregate size was quantified for all aggregates within the frame at the 36 h time point of each kinetic seeding curve, and averaged within each frame. Each point represents the average aggregate size per field, per well, with a significant increase in aggregate size initiated by rTg4510 brain extract (*P* < 0.0001) compared using a parametric Student’s unpaired *t*-test. This experiment was independently replicated three times with reproducible results. (**D**) Aggregate circularity was quantified for all aggregates within the frame at the 36 h time point of each kinetic seeding curve, and averaged within each frame. Circularity was calculated as 4π*(aggregate area)/(aggregate perimeter).^2^ The rTg4510 aggregate circularity was significantly lower than sporadic Alzheimer’s disease aggregate circularity (*P* < 0.0001) in which each data point represents average circularity in a field in a well. This experiment was independently replicated three times with reproducible results. (**E**) The average aggregate size was plot against the initial protein concentration added to inoculate the biosensor assay. There was no correlation between the initial, inoculating protein concentration and the average aggregate size (*R*^2^ = 0.0077). (**F**) The average aggregate circularity was plot against the initial protein concentration added to inoculate the biosensor assay. There was a slight correlation between the inoculating protein concentration and the aggregate circularity (*R*^2^ = 0.3837). (**G**) Average aggregate size for each patient case at the 36 h mark. (**H**) Average aggregate circularity for each patient case at the 36 h mark. (**I**) The average aggregate size for each patient case at 36 h was found to be uncorrelated with the magnitude of the plateau phase for that case (*r* = 0.158). (J) The average aggregate size for each patient case at 36 h was found to be somewhat correlated with the slope of the growth phase for that case (*r* = 0.3463). (K) The average aggregate circularity for each patient case at 36 h was found to be uncorrelated with the magnitude of the plateau phase for that case (r = 0.184). (**L**) The average aggregate circularity for each patient case at 36 h was found to be more strongly with the slope of the growth phase for that case (*r* = 0.4401).

Several reports have found differences in the morphology of the formed aggregates in this system when incubating tau from different origins.^10^^,^^11^ We analysed in our system the aggregate morphology, reported here as average size and circularity of aggregates, as an alternative endpoint of the kinetic seeding analysis. Aggregate size was found to change as a function of time, increasing during the lag phase and the growth phase, and then staying more constant during the last twelve hours, once the plateau phase is reached, both for Alzheimer’s disease cases and the rTg4510 seeds (Fig. 4A). Further analysis of aggregate size variation over the last twelve hours of seeding is shown for each patient case in Table 2. Given that aggregate morphology does not seem to change in the last part of the assay, we compared aggregate morphology for these cases at the 36-hour timepoint during the plateau phase. We first compared the morphology of aggregates formed by incubating with sporadic Alzheimer’s disease versus rTg4510 brain extracts, as these are two strains known to be conformationally different.^12^^,^^15^ As expected, we observed a significant difference in aggregate morphology, with the use of rTg4510 seeds (which are composed of P301L mutant tau) as compared to seeds from the sporadic Alzheimer’s disease cases, as can be seen in the photomicrographs (Fig. 4B) in which the rTg4510-initiated aggregates are both significantly larger (Fig. 4C, P < 0.0001) and significantly less circular (Fig. 4D, P < 0.0001). These data reinforce the idea that this kinetic time-course of aggregation analysis reveals information about different tau strains. Aggregate size was not dependent on the concentration of inoculating tau seeds when using rTg4510 brain lysate (Fig. 4E, *R*^2^ = 0.0077), though there was some variation of the circularity with concentration of inoculating tau seed (Fig. 4F, *R*^2^ = 0.3837), however, this association was not as strong as the variation of the lag phase length and plateau phase magnitude (Fig. 2B and D).

**Table 2:** Variation in aggregate size during the last 12 h of the seeding assay across all patient cases of sporadic Alzheimer’s disease. The average, standard deviation and coefficient of variation of aggregate size (computed as the ratio of the standard deviation to the mean) are reported. For all cases, the coefficient of variation is less than 0.15 (and for all but three cases, the coefficient of variation is less than 0.1), indicating little variation in the aggregate size in the last 12 h of the seeding assay.

Alzheimer’s disease Case	Average aggregate size in last 12 h (µm^2^)	Standard deviation of aggregate size in last 12 h	Coefficient of variation of aggregate size in last 12 h
Positive control	6.38	0.14415	0.02258
1	4.56	0.65323	0.14341
2	5.52	0.75611	0.13708
3	6.10	0.5747	0.09419
4	5.10	0.55487	0.10875
5	6.39	0.56497	0.08835
6	5.35	0.55318	0.10342
7	6.19	0.1975	0.03188
8	7.14	0.50424	0.07057
9	6.36	0.27118	0.04267
10	6.85	0.2485	0.03626
11	7.00	0.26051	0.03721
12	6.02	0.20612	0.03425
13	7.32	0.29073	0.03974
14	6.32	0.32203	0.05096
15	6.21	0.2775	0.04467
16	6.46	0.10479	0.01623
17	7.09	0.41882	0.05905
18	7.41	0.17647	0.02382
19	7.33	0.30151	0.04115
20	6.53	0.10405	0.01593
21	7.12	0.1274	0.0179
22	7.08	0.1789	0.02527
23	5.29	0.722	0.13639
24	6.08	0.36125	0.05937
25	6.65	0.21755	0.03272
26	7.59	0.1901	0.02503
27	7.36	0.1304	0.01771
28	6.94	0.1728	0.02491
29	7.52	0.16775	0.02229
30	7.35	0.19111	0.02601
31	6.73	0.19316	0.02872

We next tested whether these morphological characteristics varied across cases of sporadic Alzheimer’s disease. Across the 32 patients of our cohort, we found that there were quantitatively detectable differences in the morphology of the aggregates across patients consistent with our previous observation of tau heterogeneity in this cohort.^17^ Importantly, neither size nor circularity were correlated with the magnitude of the plateau phase of the case (Fig. 4G/I, *P* = 0.3840; Fig. 4H/K, *P* = 0.3160), suggesting that the differences are unrelated to the amount of tau seeds or the amount of seeding-competent tau present in the sample, as confirmed by the results of the dose response curve (Fig. 4E and F). The aggregate size was near-significantly negatively associated with the slope of the growth phase of the curve (*P* = 0.0564, Fig. 4J), reinforcing the idea that both of these parameters are independent of the number of seeds and instead related to the aggregation phenomenon. The aggregate circularity was significantly positively associated with the slope of the growth phase (*P* = 0.0132, Fig. 4L). These results suggest that while differences in aggregate morphology among Alzheimer’s disease cases are generally not evident from simple observation, quantitative metrics may prove a useful tool in studying aggregate strains moving forward.

**Figure 5:**
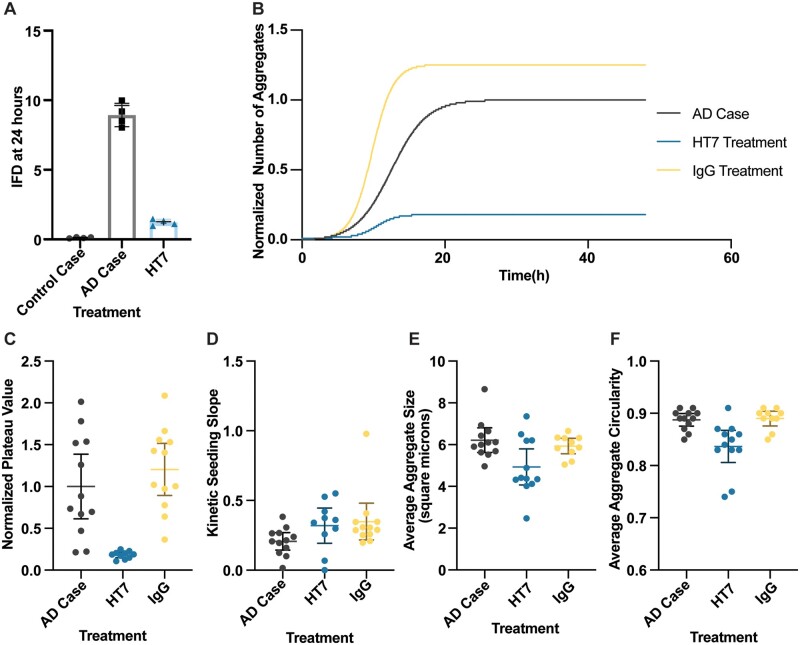
**Tau seeding kinetics after antibody reduction.** (**A**) Seeding was initiated either using seeds from the positive control Alzheimer’s disease case brain extract^31^ or seeds co-incubated with a tau antibody with a mid-domain epitope (HT7). Aggregates were quantified 24 h after the seeds were added to the biosensor cells via flow cytometry as the integrated FRET density (IFD). Antibody co-incubation significantly reduced the seeding in 4 individual experiments (*P* < 0.0001), compared using a parametric Student’s unpaired *t*-test. (**B**) Seeding was initiated either with Alzheimer’s disease Case 21 brain extract or the extract co-incubated with antibody. Aggregate count was averaged across fields in each well and normalized to the magnitude of the plateau phase of the positive control case. Co-incubation with the tau-specific antibody significantly reduced the tau seeding at the plateau whereas co-incubation with a non-specific antibody (IgG) slightly increased the tau seeding plateau. These data in this figure represent an average of three independent experiments. (**C**) The plateau phase magnitudes were normalized to the average plateau phase magnitude for the untreated Alzheimer’s disease Case. There was a significant reduction in the plateau phase magnitude when tau seeds were co-incubated with a tau specific antibody (*P* = 0.0004) whereas there was a non-significant change when tau seeds were co-incubated with a non-specific antibody (*P* = 0.3745). (**D**) The kinetic seeding slope was approximated from the Hill slope of the non-linear regression in each field in each well. There was no significant difference in the slope with co-incubation of seeds with the tau-specific antibody (*P* = 0.0715). (**E**) The average aggregate size in square micrometers was measured in each field in each well, where each point represents average aggregate size in a field. There was a significant decrease in the size of protein aggregates with co-incubation of seeds with tau-specific antibodies (*P* =0.0132) at the 36 h time point. (**F)** There was a significant decrease in aggregate circularity with co-incubation of seeds with the tau-specific antibody compared to untreated seeds (*P* = 0.0027) at the 36 h time point. All the data in this figure represent the average of three independent experimental replicates. Error bars represent s.d. of the mean.

Finally, we and others previously demonstrated that treatment of brain extracts with tau-specific antibodies reduced tau aggregation in this biosensor assay.^17^^,^^32^ We used our system to understand how this might affect the kinetics of tau aggregation. We found that co-incubation of initial tau aggregates from a human case of sporadic Alzheimer’s disease with a monoclonal antibody raised against human tau (epitope HT7, tau mid-domain amino acids 158–162) prior to inoculation of the biosensor assay reduced tau aggregation by ∼90% (*P* <0.0001; Fig. 5A). Using live-cell imaging, we found that this co-incubation significantly changed the magnitude of the plateau phase (Fig. 5B and C, P < 0.0001), but did not significantly change the slope of the seeding curve (Fig. 5D, *P* = 0.0715). The low levels of tau aggregation, however, precluded analysis of the lag phase of the curve, given that the cutoff for the lag phase was too close to the background noise of the analysis of the assay itself. The aggregate size and circularity were both reduced in the HT7 antibody co-incubation condition (Fig. 5E, P = 0.0132; Fig. 5F, *P* = 0.0027) as compared to the biosensor line inoculated with just tau seeds.

## Discussion

In this study, we modified a previously well-described tau biosensor assay (Holmes et al., 2014^8^) to study the kinetics of tau aggregation in a cellular context, revealing new properties of tau aggregates. We found that tau aggregation follows a stereotyped pattern of logistic growth (Fig. 1B) as previously suggested.^8^^,^^29^ We demonstrated that in the biosensor cells, the rate of aggregation is rapid, after a lag phase mostly dependent on the quantity of seeds introduced. This rapid growth is reminiscent of earlier *in vivo* work demonstrating that rapid aggregation can occur in the setting of ample aggregation prone substrate.^33^

In addition to the lag phase, we observed that all seeding curves reached a plateau in number of aggregates well before exhaustion of substrate. This plateau is proportional to the dose of initial, inoculated tau seeds and therefore inversely correlated to the length of the lag phase. This is further reinforced by inspection of the images showing that new seeds are not formed and existing seeds are not degraded in the plateau phase. At higher concentrations of inoculating tau seeds, the plateau phase magnitude does reach a ceiling, which we speculate reflects an exhaustion of some cellular processes necessary for tau aggregation rather than of the production of endogenous tau reporter protein (as the intracellular fluorescence not associated with aggregates, i.e. the level of diffuse fluorescent tau, is still high in the cells). Across sporadic Alzheimer’s disease cases (normalized to the same amount of total tau), the inverse correlation between the plateau and the lag phase magnitudes implies that these parameters are primarily dependent on the amount of the seeding-competent tau species inoculated.

It is important to note that we used a liposomal transfection agent to force the uptake of the seeds by the cells. Therefore, this assay intentionally does not measure the ability of seeds to be taken up by cells and delivered to the cytoplasm. Hence, the dependence of the lag phase length on tau seed concentration suggests that the lag phase length is a diffusion-limited variable, in which the intracellular accumulation of tau seeds is a random diffusive process with flux directly proportional to the concentration gradient. At higher concentrations of inoculating tau seeds, the flux of these seeds is greater and thus the time until a pathological tau seed comes into contact with the endogenous tau is reduced.

With regards to the growth phase, we found that the rate of growth in aggregate number was only slightly associated with the concentration of inoculated tau seeds (Fig. 2C and G) and was (i) unaffected by an antibody-mediated reduction in tau seeds and (ii) independent of the seeding capability across Alzheimer’s disease cases. This suggested that the slope of the growth phase is largely independent of the dosage of seeding-competent tau species. However, we could correlate this slope with the aggregates’ morphology across cases of sporadic Alzheimer’s disease suggesting that both the aggregate morphology and the slope of the growth curve reflect specific aggregation properties of the seeds. Previous reports have indeed shown that differences in aggregate morphology (reflective of different tau strains) could be reproduced to a certain extent in the aggregates produced in this *in vitro* model system^10^ reinforced here by the clear morphological differences observed between aggregates initiated by rTg4510 or sporadic Alzheimer’s disease tau seeds.^12^^,^^15^^,^^34^ While we recognize that structure of aggregates within this biosensor cell system does not represent structure of mature paired helical filaments,^35^ we think that these morphological differences are likely due to folding differences at the tau assembly level as seen in recent cryogenic electron microscopy reports^13^^,^^14^^,^^16^^,^^36^ and also to the presence of specific post-translational modifications (leading to subtle differences in conformational structures) in the original inoculating tau seeds across sporadic Alzheimer’s disease patients.^17^^,^^37^

An interesting observation is that aggregate size does not change dramatically once the plateau phase is reached, despite continued exposure to substrate, implying cellular mechanisms that can cap the growth of an aggregate. The kinetic analysis could therefore provide tools to screen for biological mechanisms, including proteostatic networks, impacting these capping mechanisms. Moreover, previous reports have shown that dividing cells do propagate aggregates to the daughters,^10^ arguing that processes that occur during cell division allow for tau aggregate fragmentation and new aggregate formation. Understanding the biology underlying this presumed fragmentation process could be accomplished with modifications of this longitudinal imaging approach.

Finally, given that all of the parameters studied here vary across Alzheimer’s disease patients, this confirms and extends our observation that different cases of Alzheimer’s disease vary both in amount of seeding-competent tau species as a fraction of amount of tau, and in specific aggregation properties of those seeds.^17^

## Abbreviations

CFP =: cyan fluorescent protein
FRET =: Förster resonance energy transfer
YFP =: yellow fluorescent protein
